# The orbitofrontal cortex functionally links obesity and white matter hyperintensities

**DOI:** 10.1038/s41598-020-60054-x

**Published:** 2020-02-19

**Authors:** Bo-yong Park, Kyoungseob Byeon, Mi Ji Lee, Se-Hong Kim, Hyunjin Park

**Affiliations:** 1 McConnell Brain Imaging Centre, Montreal Neurological Institute and Hospital, McGill University, Montreal, H3A 2B4 Canada; 2 Department of Electrical and Computer Engineering, Sungkyunkwan University, Suwon, 16419 South Korea; 3 Center for Neuroscience Imaging Research, Institute for Basic Science (IBS), Suwon, 16419 South Korea; 4 Department of Neurology, Samsung Medical Center, Sungkyunkwan University School of Medicine, Seoul, 06351 South Korea; 5 Department of Family Medicine, St. Vincent’s Hospital, Catholic University College of Medicine, Suwon, 16247 South Korea; 6 School of Electronic and Electrical Engineering, Sungkyunkwan University, Suwon, 16419 South Korea

**Keywords:** Neuroscience, Neurological disorders

## Abstract

Many studies have linked dysfunction in cognitive control-related brain regions with obesity and the burden of white matter hyperintensities (WMHs). This study aimed to explore how functional connectivity differences in the brain are associated with WMH burden and degree of obesity using resting-state functional magnetic resonance imaging (fMRI) in 182 participants. Functional connectivity measures were compared among four different groups: (1) low WMH burden, non-obese; (2) low WMH burden, obese; (3) high WMH burden, non-obese; and (4) high WMH burden, obese. At a large-scale network-level, no networks showed significant interaction effects, but the frontoparietal network showed a main effect of degree of obesity. At a finer node level, the orbitofrontal cortex showed interaction effects between periventricular WMH burden and degree of obesity. Higher functional connectivity was observed when the periventricular WMH burden and degree of obesity were both high. These results indicate that the functional connectivity of the orbitofrontal cortex is affected by the mutual interaction between the periventricular WMHs and degree of obesity. Our results suggest that this region links obesity with WMHs in terms of functional connectivity.

## Introduction

Obesity is a worldwide health problem characterized by the excessive accumulation of body fat, which leads to several comorbid conditions such as type 2 diabetes, cardiovascular disease, stroke, and various cancers^1–3^. Obesity is a multi-factorial disease affected by environmental, hereditary, and behavioral factors^3–5^. Recent studies have shown that obesity is also associated with alterations in the brain that can be explored using neuroimaging^3,6–8^.

Previous obesity-related neuroimaging studies have measured the functional connectivity of the brain using functional magnetic resonance imaging (fMRI) and found dysfunctions in cognitive control-related brain regions^3,8–11^. Specifically, they found that the frontoparietal and executive control networks responsible for cognitive- and inhibitory-controls were strongly associated with binge eating behaviors^9–11^. Structural alterations in reward and cognition-related brain regions have also been observed in people with obesity^9,12^. Collectively, these results suggest that cognitive control-related brain regions may be important in explaining the behavioral traits of obese subjects.

Relatedly, another recent neuroimaging study reported that a high burden of white matter hyperintensities (WMHs) was associated with obesity^13^. WMHs are brain lesions that show an aberrant increase in white matter intensity on fluid-attenuated inversion recovery (FLAIR) data. They are related to an increased risk of cognitive decline, dementia, and stroke^14–16^. Some research suggests that white matter vascularization is related to obesity and comorbid metabolic dysfunction^13,17–20^. However, the existing neuroimaging literature has not considered the burden of WMHs to stratify the degree of obesity. The present study aimed to address this gap in research by considering the burden of WMHs and the degree of obesity simultaneously.

Connectivity analysis is one of the representative methods to measure brain function^21,22^. In this study, we adopted a functional connectivity analysis based on graph theory to measure the strength of intrinsic connectivity in the brain^21,22^. The two fundamental factors of the analysis were nodes and edges. The graph nodes represented brain regions or networks defined using structural atlases or data-driven approaches, such as clustering or independent component analysis (ICA)^23–27^. The graph edges were defined as the strength of the connection between two different nodes^28^.

We hypothesized that WMHs and obesity jointly affect the function of cognitive control-related brain regions. In the present study, we aimed to explore changes in functional connectivity with respect to the burden of WMHs and the degree of obesity to assess their interaction effects on the brain connectome. We performed two-way analysis of variance (ANOVA) to compare functional connectivity among four groups stratified by the degree of obesity and WMH burden. The results of the study may provide novel insight into the neurological characteristics of people with both obesity and WMHs.

## Methods

### Participants

The Institutional Review Board (IRB) of Sungkyunkwan University approved the present retrospective study, which was performed in full accordance with local IRB guidelines. All participants provided written informed consent. T1-weighted, FLAIR, and resting-state fMRI (rs-fMRI) data were obtained from the UK Biobank database^29^ through application number 34613 entitled “Neuroimaging correlates of obesity.” Among 13,718 participants, 91 did not have waist circumference, hip circumference, or body mass index data, while 29 lacked T1-weighted, FLAIR, or rs-fMRI data and 13,416 did not have WMHs. These participants were excluded. Ultimately, 182 participants were included in the present study. Detailed demographic information is reported in Table 1.

**Table 1:** Demographic information of the study participants.

Information	Mean (SD)
Age (years)	55.21 (7.16)
Sex (male:female)	100:82
Waist circumference (cm)	88.15 (11.68)
Hip circumference (cm)	103.21 (7.67)
Waist-hip ratio	0.85 (0.08)
Body mass index (kg/m^2^)	26.80 (4.10)
Healthy weight: Overweight: Obese	65:83:34
Total WMH volume (mm^3^)	3145.21 (3227.76)
Deep WMH volume (mm^3^)	386.31 (744.17)
Periventricular WMH volume (mm^3^)	2758.91 (2928.18)

SD, standard deviation; WMH, white matter hyperintensity.

### MRI data acquisition

All imaging data were acquired using a 3T Siemens Skyra scanner. The imaging acquisition parameters of the T1-weighted data were as follows: voxel size = 1 mm^3^; repetition time (TR) = 2,000 ms; inversion time (TI) = 880 ms; matrix size = 208 × 256 × 256. The FLAIR data were acquired using the following imaging parameters: voxel size = 1.05 × 1 × 1 mm^3^; TR = 5,000 ms; TI = 1,800 ms; matrix size = 192 × 256 × 256. The rs-fMRI data were obtained with the following imaging parameters: voxel size = 2.4 mm^3^; TR = 735 ms; echo time (TE) = 39 ms; flip angle = 52°; matrix size = 88 × 88 × 64; number of volumes = 490.

### Data preprocessing

The UK Biobank database provided preprocessed imaging data through the FMRIB Software Library (FSL) software^30,31^. To process the T1-weighted data, gradient distortion corrected data were registered onto the Montreal Neurological Institute (MNI) standard space. Non-brain tissues were then removed via inverse warping of the brain mask of the MNI standard space to the native T1-weighted space. Next, these skull-removed T1-weighted data were segmented into three tissues: cerebrospinal fluid, gray matter, and white matter. Finally, the magnetic field inhomogeneity was corrected. To process the FLAIR data, gradient distortion corrected data were registered onto the T1-weighted data to extract the brain, and the magnetic field inhomogeneity was corrected. To process the rs-fMRI data, gradient distortions and head motions were corrected, intensity normalization of the entire 4D volume was applied, as was high-pass temporal filtering with a sigma of 50 s was applied. Nuisance variables were removed using FMRIB’s ICA-based X-noiseifier (ICA-FIX) approach^32^.

### Specification of WMHs

The UK Biobank database provided WMH masks computed from the Brain Intensity Abnormality Classification Algorithm (BIANCA) software^33^, which is a supervised machine learning algorithm^34^ of k-nearest neighbor that uses voxel- and patch-based intensity values of FLAIR data, as well as spatial coordinates of MNI standard space. Leave-one-out cross-validation was used for the training and test procedures. BIANCA produced a probability map of WMHs, which was then thresholded and binarized with a value of 0.5. However, BIANCA sometimes fails to capture small, deep WMHs^35^; hence, we manually adjusted the WMH masks computed by BIANCA. These adjusted WMHs were further classified into deep and periventricular WMHs. Deep WMHs showed hyperintensities with variable round- or oval-shaped clusters in the white matter on FLAIR images^36^. Periventricular WMHs showed hyperintensities along the walls of the ventricles, appearing as small caps, thin rims, or confluent lesions on FLAIR images^37,38^. The WMHs were manually annotated by two investigators (M.J.L., with 9 years’ experience in clinical neurology, and B.P., with 7 years’ experience in neuroimaging analysis). Inter-observer reliability was assessed using the dice coefficient, which yielded values of 0.93 (95% confidence interval [CI]: 0.89–0.97) for total WMHs, 0.93 (95% CI: 0.90–0.96) for deep WMHs, and 0.92 (95% CI: 0.86–0.97) for periventricular WMHs.

### Functional connectivity analysis

Functional connectivity analysis was performed using a multi-scale approach. First, a large-scale network-level analysis was performed. Graph nodes (i.e., brain networks) were defined by group ICA^23,24^, which was performed on the temporally concatenated, preprocessed rs-fMRI data across all subjects using the MELODIC function in the FSL software^31^. The number of independent components (ICs) was automatically determined based on probabilistic principal component analysis^23,24,39^. The investigators removed noise ICs by visual inspection and by comparing them with the pre-defined resting-state networks (RSNs); ICs with a cross-correlation value below 0.2 were considered noise ICs. Secondly, a node-level analysis was performed using the automated anatomical labeling (AAL) and Brainnetome atlases to assess consistency across different parcellation schemes^26,27^. Graph nodes were pre-defined regions of the atlas. In both the large-scale network- and node-level analyses, the mean time series of the rs-fMRI data was extracted for each graph node (i.e., brain network/region). Pearson’s correlation was then calculated for the time series between the two different nodes. The correlation coefficients were soft-thresholded to satisfy scale-free topology using the following formula: {(r + 1)/2}^β^, where r is the correlation coefficient and β is the scale-free index, which was set to six^40,41^. The soft-thresholded correlation coefficients were then transformed into z-values using Fisher’s r-to-z transformation. Degree centrality, a graph measure that estimates the strength of the functional connectivity at a given node, was calculated by summing all edge weights connected to a given node^21,22^. Degree centrality values were adjusted for age and sex.

### Group comparison

The degree centrality values of the brain networks/regions were compared among the four groups stratified by burden of WMHs and degree of obesity: (1) low WMH burden, non-obese (lw-no); (2) low WMH burden, obese (lw-o); (3) high WMH burden, non-obese (hw-no); (4) high WMH burden, obese (hw-o) (Table 2). The cutoff value between high and low WMH burden was the median WMH volume. There is no consensus regarding how to distinguish high and low WMH burden. Thus, in the present study, we stratified the groups using a data-driven approach based on the median WMH volume from all subjects (n = 182). However, further studies are needed to validate the usage of median WMH volume as the cutoff. To explore the differences between deep and periventricular WMHs, the median deep and periventricular WMH volumes were considered in addition to the total WMH volume. The waist-hip ratio was used instead of body mass index to stratify the obese and non-obese groups because it is a well-defined measure of metabolically unhealthy obesity, which is strongly associated with obesity-related complications such as diabetes and cardiovascular diseases^42–46^. The obese groups had a waist-hip ratio larger than 0.9 in males and 0.85 in females^47^. Two-way ANOVA was applied to the factors of WMH burden (low vs. high) and degree of obesity (non-obesity vs. obesity) to assess both the main effects and the interaction effects between WMH burden and degree of obesity. Both F- and p-values were calculated. Post-hoc analysis was performed using the two-sample t-test; both T- and p-values were calculated. All p-values were corrected using the false discovery rate (FDR) suggested by Benjamini and Hochberg^48^. The p-values of the two-way ANOVA were corrected for the number of brain regions, while those of the post-hoc analysis were corrected for the number of group comparisons.

**Table 2:** Demographic information of the study participants in each group.

Criteria	Information	lw-no	lw-o	hw-no	hw-o	P-value
Total WMHs	Number of subjects	53	38	54	37	N/A
	Age (years)	53.62 (7.81)	57.34 (5.87)	53.44 (7.10)	57.89 (6.24)	0.0028
	Sex (male:female)	15:38	22:16	15:39	30:7	<0.001*
	Waist circumference (cm)	82.55 (8.35)	95.74 (8.48)	81.11 (8.98)	98.68 (9.84)	<0.001
	Hip circumference (cm)	102.32 (6.97)	104.26 (8.47)	102.49 (8.21)	104.46 (6.93)	0.1546
	Waist–hip ratio	0.81 (0.06)	0.92 (0.04)	0.79 (0.06)	0.94 (0.05)	<0.001
	Body mass index (kg/m^2^)	25.62 (3.57)	27.86 (4.25)	25.51 (3.73)	29.31 (3.88)	<0.001
	WMH volume (mm^3^)	1094.43 (498.13)	1204.87 (474.26)	5142.06 (3576.13)	5161.30 (3555.23)	<0.001
Deep WMHs	Number of subjects	54	37	53	38	N/A
	Age (years)	54.35 (7.64)	57.41 (6.02)	52.70 (7.18)	57.82 (6.10)	<0.001
	Sex (male:female)	15:39	24:13	15:38	28:10	<0.001*
	Waist circumference (cm)	82.07 (9.29)	97.14 (8.89)	81.56 (8.06)	97.24 (9.67)	<0.001
	Hip circumference (cm)	102.07 (7.55)	104.54 (8.64)	102.75 (7.67)	104.18 (6.77)	0.1526
	Waist-hip ratio	0.80 (0.06)	0.93 (0.05)	0.79 (0.06)	0.93 (0.05)	<0.001
	Body mass index (kg/m^2^)	25.43 (3.87)	28.72 (4.47)	25.69 (3.41)	28.43 (3.78)	<0.001
	WMH volume (mm^3^)	54.11 (32.72)	45.22 (35.88)	731.28 (1002.09)	709.34 (860.22)	<0.001
Periventricular WMHs	Number of subjects	54	37	53	38	N/A
	Age (years)	53.67 (7.80)	57.52 (6.10)	53.41 (7.14)	57.71 (6.02)	0.0039
	Sex (male:female)	16:35	24:16	14:42	28:7	<0.001*
	Waist circumference (cm)	82.49 (8.53)	95.88 (8.41)	81.21 (8.81)	98.69 (10.01)	<0.001
	Hip circumference (cm)	102.08 (6.88)	104.38 (8.28)	102.71 (8.22)	104.34 (7.09)	0.1423
	Waist-hip ratio	0.81 (0.06)	0.92 (0.04)	0.79 (0.05)	0.94 (0.05)	<0.001
	Body mass index (kg/m^2^)	25.58 (3.54)	27.86 (4.12)	25.54 (3.75)	29.38 (4.01)	<0.001
	WMH volume (mm^3^)	914.47 (410.90)	1033.83 (431.33)	4417.11 (3270.81)	4764.91 (3256.12)	<0.001

^*^Chi-square test. The lowest p-value was reported by comparing the four groups.

lw, low WMH burden; hw, high WMH burden; no, non-obese; o, obese; N/A, not available.

## Results

### Large-scale network-level analysis

**Figure 1:**
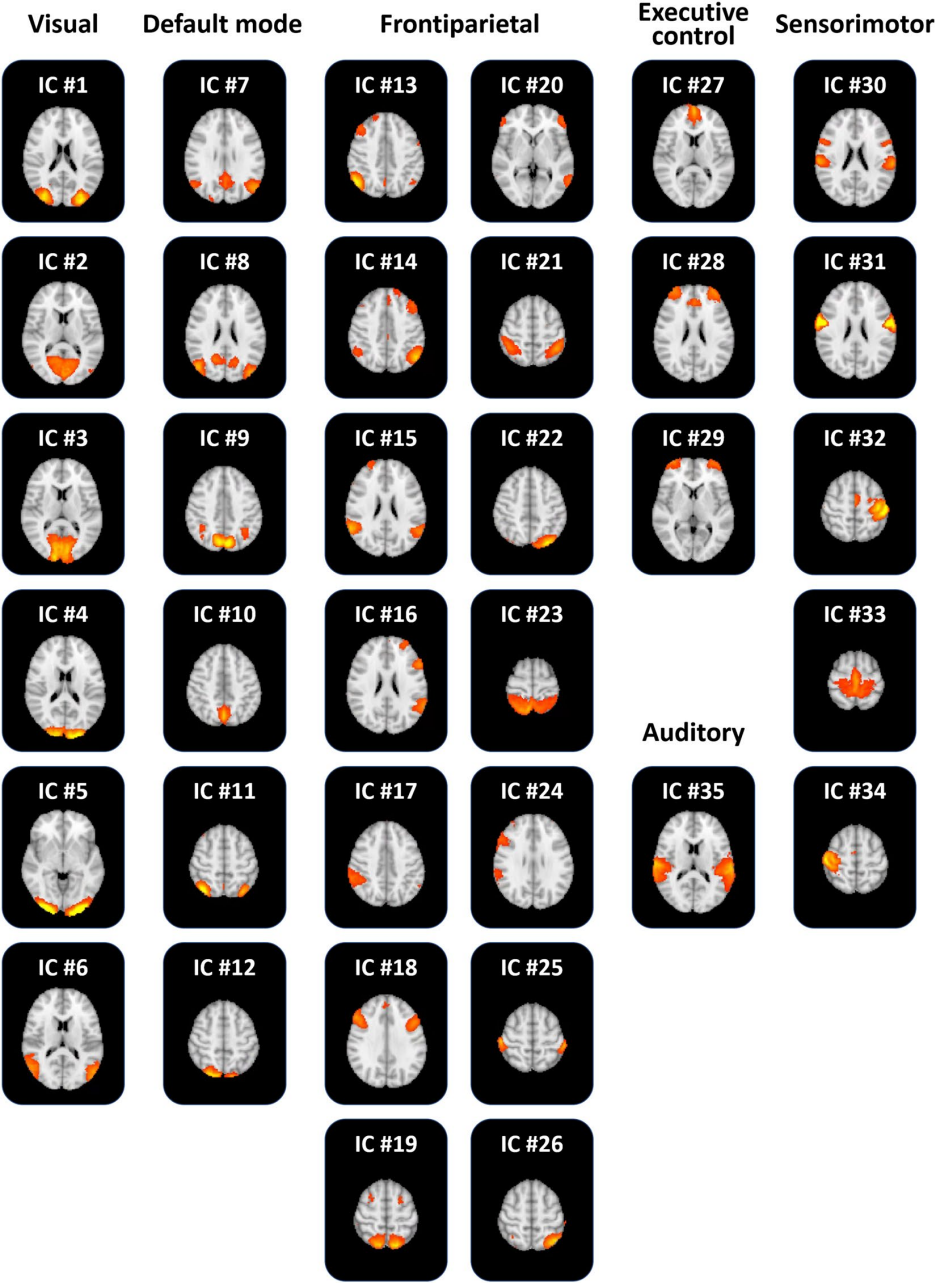
The 35 functionally interpretable independent components (ICs).

**Figure 2:**
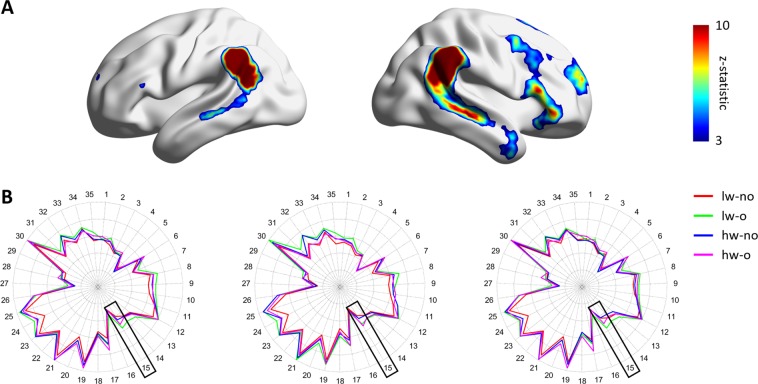
Between-group comparison at the large-scale network-level. (**A**) Frontoparietal network (IC #15) mapped onto the brain surface. The color map represents the effect size of the z-statistic values derived from the FSL software. (**B**) Degree centrality values of all independent components (ICs) in each group. The black boxes represent the frontoparietal network (IC #15). Brain images were visualized using the BrainNet Viewer^49^. lw, low WMH burden; hw, high WMH burden; o, obese; no, non-obese.

Group ICA was performed to define large-scale brain networks. Forty-two ICs were automatically generated, and seven noise ICs were excluded. The 35 functionally interpretable ICs (mean correlation with RSN: 0.31, standard deviation [SD]: 0.14) were considered as graph nodes (Fig. 1). ICs 1–6 were visual networks, 7–12 were default mode networks, 13–26 were frontoparietal networks, 27–29 were executive control networks, 30–34 were sensorimotor networks, and 35 was an auditory network. Two-way ANOVA was performed to assess the interaction effects between WMH burden and degree of obesity using the degree centrality values. No network showed significant interaction effects. However, a significant main effect of degree of obesity was found in the frontoparietal network (IC #15; Fig. 2; F_(1,178)_ = 13.471, p < 0.001 for total WMHs; F_(1,178)_ = 13.299, p < 0.001 for deep WMHs; F_(1,178)_ = 12.823, p < 0.001 for periventricular WMHs).

### Node-level analysis

**Figure 3:**
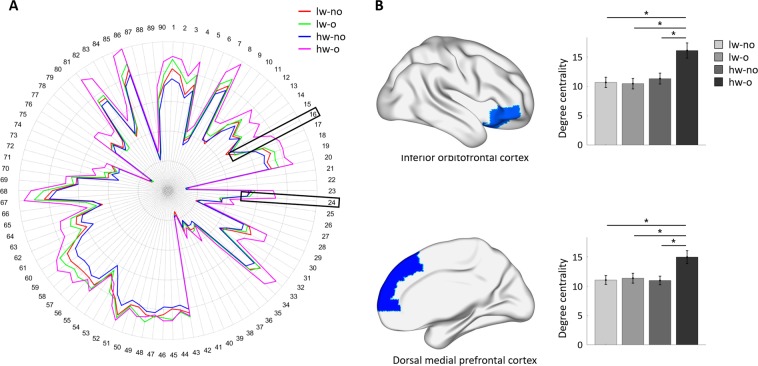
Between-group comparison at the node-level using the AAL atlas when periventricular white matter hyperintensities (WMHs) were considered. (**A**) Degree centrality values of all regions in each group. The inferior orbitofrontal cortex (region #16) and the dorsal medial prefrontal cortex (region #24) are represented with black boxes. (**B**) The three identified regions, with corresponding degree centrality values in all groups. Significant differences are shown with asterisks. Brain images were visualized using the BrainNet Viewer^49^. lw, low WMH burden; hw, high WMH burden; o, obese; no, non-obese.

**Figure 4:**
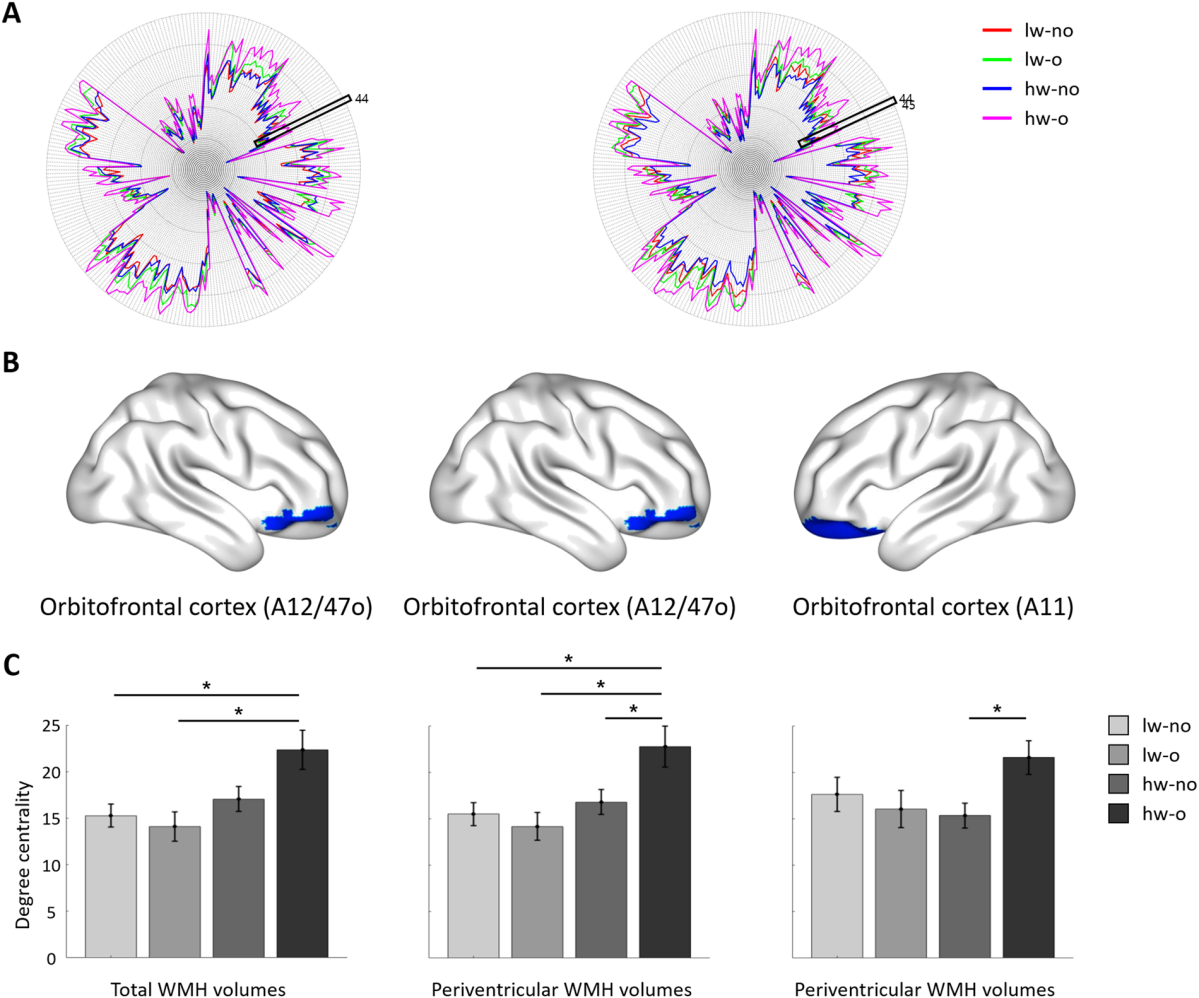
Between-group comparison at the node-level using the Brainnetome atlas. (**A**) Degree centrality values of all regions for each group when total (left) and periventricular WMHs (right) were considered. The black boxes represent the orbitofrontal cortex (A12/47o and A11). (**B**) The identified brain regions when total (left) and periventricular WMHs (middle and right) were considered. (**C**) Degree centrality values of the identified regions in all groups. Significant differences are shown with asterisks. Brain images were visualized using the BrainNet Viewer^49^. lw, low WMH burden; hw, high WMH burden; o, obese; no, non-obese.

A node-level analysis using AAL and the Brainnetome atlas was performed to assess the interaction effects between WMH burden and degree of obesity at a finer level. Using the AAL atlas, significant interaction effects were found in the right orbitofrontal cortex (F_(1,178)_ = 5.646, p = 0.0190) and right dorsal medial prefrontal cortex (Fig. 3; F_(1,178)_ = 4.344, p = 0.0390) if periventricular WMHs were considered (Fig. 3). The post-hoc analysis revealed higher degree centrality values in the hw-o group than in the other groups for both the orbitofrontal and dorsal medial prefrontal cortices (Table 3). No interaction effects were identified when total or deep WMHs were considered. To assess the consistency of the results across different parcellation schemes, we derived additional results using the Brainnetome atlas, which was defined using multimodal (i.e., structural and functional) connectivity information^27^. When total WMHs were considered, a significant interaction effect was found in the right orbitofrontal cortex (A12/47o; Fig. 4; F_(1,178)_ = 4.381, p = 0.0380). The post-hoc analysis revealed higher degree centrality values in the hw-o group than in the lw-no and lw-o groups (Table 4). No significant interaction effects were observed if deep WMHs were considered. For periventricular WMHs, the right orbitofrontal cortex (A12/47o; F_(1,178)_ = 5.659, p = 0.0180) and left orbitofrontal cortex (A11; F_(1,178)_ = 4.979, p = 0.0270) showed significant interaction effects. In the right orbitofrontal cortex (A12/47o), post-hoc analysis exhibited higher degree centrality values in the hw-o group than in the other groups, while in the left orbitofrontal cortex (A11), the hw-o group showed higher degree centrality values than the hw-no group (Table 4). The results derived from both the AAL and Brainnetome atlases consistently showed significant interaction effects and post-hoc results in the orbitofrontal cortex, indicating that this is a key region linking WMH and obesity.

**Table 3:** Post-hoc analysis at the node-level using the AAL atlas when the periventricular WMHs were considered. Significant results are reported in bold italics.

Region	Group comparison	Post-hoc analysis		
		DOF	T-value	p-value
R. Inferior orbitofrontal cortex	lw-no vs. lw-o	89	0.3189	0.7506
	lw-no vs. hw-no	105	−0.5436	0.7054
	***lw-no vs. hw-o***	***84***	***−3.3484***	***0.0037***
	lw-o vs. hw-no	94	−0.8293	0.6136
	***lw-o vs. hw-o***	**73**	***−3.5445***	***0.0037***
	***hw-no vs. hw-o***	***89***	***−2.8019***	***0.0125***
R. Dorsal medial prefrontal cortex	lw-no vs. lw-o	89	−0.2030	0.9600
	lw-no vs. hw-no	105	0.0502	0.9600
	***lw-no vs. hw-o***	84	***−2.8874***	***0.0148***
	lw-o vs. hw-no	94	0.2505	0.9600
	***lw-o vs. hw-o***	***73***	***−2.5702***	***0.0244***
	***hw-no vs. hw-o***	***89***	***−2.9481***	***0.0148***

lw, low WMH burden; hw, high WMH burden; o, obese; no, non-obese; DOF, degrees of freedom.

**Table 4:** Post-hoc analysis at the node-level using the Brainnetome atlas.

Criteria – Region	Group comparison	Post-hoc analysis		
		DOF	T-value	p-value
Total WMHs – R. Orbitofrontal cortex (A12/47o)	lw-no vs. lw-o	89	0.7045	0.4854
	lw-no vs. hw-no	105	−0.7001	0.4854
	***lw-no vs. hw-o***	***88***	***−3.0994***	***0.0079***
	lw-o vs. hw-no	90	−1.2965	0.2970
	***lw-o vs. hw-o***	***73***	***−3.3121***	***0.0079***
	hw-no vs. hw-o	89	−2.466	0.0312
Periventricular WMHs – R. Orbitofrontal cortex (A12/47o)	lw-no vs. lw-o	89	0.7045	0.4854
	lw-no vs. hw-no	105	−0.7001	0.4854
	***lw-no vs. hw-o***	***84***	***−3.0994***	***0.0079***
	lw-o vs. hw-no	94	−1.2965	0.2970
	***lw-o vs. hw-o***	***73***	***−3.3121***	***0.0079***
	***hw-no vs. hw-o***	***89***	***−2.466***	***0.0312***
Periventricular WMHs – L. Orbitofrontal cortex (A11)	lw-no vs. lw-o	89	0.5863	0.6710
	lw-no vs. hw-no	105	1.0159	0.4680
	lw-no vs. hw-o	84	−1.4756	0.2876
	lw-o vs. hw-no	94	0.3010	0.7641
	lw-o vs. hw-o	73	−2.0591	0.1291
	***hw-no vs. hw-o***	***89***	***−2.8289***	***0.0346***

lw, low WMH burden; hw, high WMH burden; o, obese; no, non-obese; DOF, degrees of freedom.

Significant results are shown in bold italics.

## Discussion

In the present study, we used a multi-scale approach to explore differences in functional connectivity associated with WMH burden and degree of obesity. We found that in the frontoparietal network, the orbitofrontal cortex was jointly associated with WMH burden and degree of obesity, while the parietal networks were only related to the degree of obesity. These results indicate that the orbitofrontal cortex is a key region linking WMH and obesity, and that the frontoparietal network is primarily related to the degree of obesity, but not WMH burden.

Frontoparietal network is involved in the cognitive control system, controlling inhibitory behaviors^50–52^. It sends inhibitory signals to the limbic area to suppress the feeling of hunger^53^. Previous studies have demonstrated that perturbed connections between the prefrontal cortex, striatum, and limbic regions disrupted the balance between cognition and reward systems, leading to binge eating disorders^54–57^. In our previous studies, we reported that dysfunction in the frontoparietal network was associated with obesity via a mechanism involving disinhibited eating behaviors, and that participants with such dysfunction had concerns about their eating habits, shape, and weight^9–11^. These studies collectively suggest that the frontoparietal network is crucial in explaining the behavioral traits of individuals with obesity, and our current findings largely corroborate these results, linking obesity with altered functional connectivity in the frontoparietal network.

At the finer node-level, we observed that both WMH burden and degree of obesity affected functional connectivity in the orbitofrontal cortex, which controls the reward system by encoding food-related reward responses and inducing the feeling of hunger^53,58–62^. In addition, the orbitofrontal cortex is involved in the cognitive control system of inhibitory processing^53,58^. One previous study showed that the dysfunctional inhibitory control that leads to overeating is related to an increased demand for reward processing, suggesting links between the reward and cognitive control systems^61^. These studies collectively indicate that the identified regions are related to cognitive function, which is highly associated with WMHs^14–16^. Our results suggest that increased WMH burden in obesity affects altered functional connectivity in the orbitofrontal cortex that controls response inhibition and reward processing. We believe that such changes may contribute to further aberrant eating behavior, though further studies need to confirm this hypothesis.

The most important risk factors for WMHs are age and hypertension^63–66^. Almost half of the population has WMHs in midlife, and the incidence increases with age^63–66^. Previous studies have reported that the prevalence of WMHs in elderly subjects is associated with cognitive and functional impairment^65^, as well as with cortical thinning in the frontal areas of individuals with mild cognitive impairment and dementia^66^. Such changes lead to altered executive functions. These studies collectively indicate that the WMH burden is linked to cognitive decline with aging. Previous studies have demonstrated that obesity is related to a decline in cognitive function^67,68^. Using diffusion tensor imaging, Zhang *et al*. found that visceral obesity was associated with executive functions^68^, while Fitzpatrick *et al*. observed that mid-life obesity was strongly related to an increased risk of dementia^67^. Another previous study found that visceral obesity, one of the major risk factors of cognitive decline^69,70^, was associated with the presence of WMHs^13^. Taken together, these studies suggest that cognitive control-related function may be affected by both WMH burden and degree of obesity. To our knowledge, the current study was the first to link WMH burden with degree of obesity in terms of functional connectivity. The results may provide insight into cognitive function in individuals with both obesity and WMHs. To validate our findings, future studies should explore the associations among WMH burden, degree of obesity, eating behaviors, and cognition-related clinical scores such as Mini-Mental State Examination (MMSE) and Clinical Dementia Rating (CDR). These parameters could not be compared in our current study because the UK Biobank database does not provide these clinical scores. Further longitudinal studies are also required to fully validate how changes in cognitive function are related to WMH burden and degree of obesity.

Our present study had several limitations. First, obesity is affected by many factors, including hormones and toxins other than those listed in Table 1. Hormones such as leptin and ghrelin convey appetite-related information to the hypothalamus, regulating eating behavior^71,72^. These factors should be controlled for in the compared groups. However, we could not do so in the present study because we downloaded the data retrospectively from the UK Biobank database. Second, although there are many centrality measures, we only used degree centrality to quantify complex brain networks because it is a convenient graph measure to associate brain imaging with obesity^9,11^. Different graph centrality measures quantify different aspects of the brain network, and future works should explore these^73^. Third, the number of participants was relatively small compared to that in previous studies^13^. We had difficulty obtaining large-scale T1-weighted, FLAIR, and rs-fMRI data with sufficient WMHs. Future studies with larger samples are necessary to fully validate the results of our current study. In a similar vein, future studies should consider a multi-center/multi-database approach to verify whether the results can be generalized to many different cohorts. Lastly, total, deep, and periventricular WMHs are not independent of one another. Studies are indeterminate regarding the distinct pathophysiological backgrounds of deep and periventricular WMHs^65,66,74–78^. Periventricular WMHs show a strong association with age, hypertension, and cognitive decline, and are primarily observed in middle-aged and elderly individuals^65,66,74,75^, while deep WMHs are prevalently observed in young adults with migraine^65,76,77^. The present study was exploratory and did not aim to confirm any hypothesis regarding which WMH types are more related to obesity. Thus, we considered two subtypes of WMH (i.e., deep and periventricular), as well as total WMHs. Further studies should confirm the clear relationship between WMH subtypes and obesity.

The present study explored differences in brain functional connectivity with respect to WMH burden and degree of obesity. Among the frontoparietal network, which is largely associated with cognitive control function in individuals with obesity, the orbitofrontal cortex was identified as the key region involved in the link between WMH and obesity. The results of our study may provide a rationale for exploring the link between WMH burden and cognitive control functions in people with obesity.

## Data Availability

The imaging and phenotypic data are available from the UK Biobank repository (https://www.ukbiobank.ac.uk/). Interested researchers should contact the database administrator to request access to the data.
